# Lallemand on the Psychology of the Reproductive Functions

**Published:** 1849-01-01

**Authors:** 


### THE JOURNAL

OF

### PSYCHOLOGICAL MEDICINE

AND

### MENTAL PATHOLOGY.

JANUARY 1, 1849.

### Analytical Reviews.

ART. I.—*Des Pertes Seminales Involontaires.* Par M. LALLEMAND, Professeur à la Faculté de Médécine de Montpellier. Paris.

*A Practical Treatise on the Causes, Symptoms, and Treatment of Spermatorrhœa.* By M. LALLEMAND. Translated by HENRY J. McDOUGALL, Esq., Member Roy. Col. Surg., England; Fellow of the Royal Medico-Chir. Soc.; &c. &c. London, 1847.

THERE is extant in the world much unwholesome morality: there is much substitution of words for things: much false delicacy which is miscalled virtue: much traffic in

"The False Commerce of Truth Unfelt:"

much conventional lying: much conventional dissimulation. For want of the reverse of all this many fine minds are overthrown. There is nothing which *is*, which has actuality of existence, that should not be fathomed, and whose rocks and quicksands should not be placed, as in an unfolded map, conspicuously in sight. Upon subjects on which neither Religion nor Science disdain to treat, correct information should be diffused. Vindications of, and apologies for, entering upon a subject like that which we propose to elucidate are but concessions to weak minds. It lies within the scope of our volition to shun many of the first approaches to insanity; nay, even, although the first steps into error are those which are most easily retraced, to extricate others and ourselves from its labyrinths when deeply involved in them. Into these labyrinths we are usually misled by some of those passions and temptations which, not peculiar to a few, are common to our whole species. We cannot know too much: and minds may be capable and yet unenlightened: hearts human and yet unawakened. Poetically, if not literally speaking, it certainly may be said with as much truth as Milton sung of "man's first disobedience" and the fatal "tree" of "the forbidden fruit," that we have ventured upon the treatment of

"Things unattempted yet in prose or rhyme."

It is true that what Dante and others were to Milton, Lallemand has been to us; while we but profess to be to him as Dun Scotus and Bellarmine were to Aristotle, in so far as our treatise bears relation to natural science only. It is our purpose, however, to trespass over those limits of natural science which Lallemand has assigned himself, and to treat the subject in a manner more strictly and severely ethical, inasmuch as we can no otherwise trace out, link by link, the chain which connects it with insanity. It is a subject on which even sane minds are apt to entertain many misconceptions. There are psychological mysteries which it lies within the power of pathology to elucidate, and which would, without its aid, remain obscure. There have frequently been witnessed deviations from the perfectly correct in conduct, and amiable in manners; exhibitions of petulance of temper and trespasses against the minor moralities; to account for which, upon a *post mortem* examination, there have been discovered traces of painful and perhaps previously unsuspected organic disease. Among our currently nomenclatured diseases are some which peculiarly tend to generate gloom, and even in severe or long protracted cases, to incite to suicide. There are forms of gastric, hepatic, and cerebral disease which display these or like tendencies. Sometimes it is rather ill temper that is induced, as by attacks of gout. Anxiety of expression in the countenance is a symptom of enteritis, which, although having a physical origin, has not a physical character only, but implicates the condition of the mind. The mind in each of the varied forms of febrile excitement takes the peculiar course of wandering, surrounds itself with those peculiar groups of hallucinations, which characterise the existing state of the brain and sensory system. At the same time it may be observed, that the mind of the patient, individually, very considerably determines the mode in which various morbid states of the brain are manifested; and will be found, in a greater or less degree, unless when torpor and incapacity are superinduced, to vindicate its own idiosyncrasy. While under excitement, it frequently throws off such gigantic shadows of portions of its being as the oxyhydrogen microscope brings into view of the minuter textures of natural objects. We obtain glimpses of the very *infusoria*, so to speak, which are engendered in the reason and imagination of the patient. There are, on the other hand, diseases, and these of a fatal character, which, during the most part of their course, do not disturb the temper or trouble the mind. Consumption, not always indeed, but frequently and commonly, deals in these respects very gently with her victims. Investing them as with the hues of perpetual youth, she leads them to the altar crowned with garlands. Not till their near approach to the sacrificial flame, does the bright eye lose its lustre, the hectic flush give place to paleness—do the hues fade, the garlands wither. They gradually, though still and evermore attended by Hope, become less and less tenacious of existence: they are gently weaned from the things of time and sense, and from the love of life: their hopes in life are displaced by hopes of the life beyond this life. We regard their fate almost with envy. After bereaved relatives have passed through the first bitterness of sorrow for their loss, their reminiscences of those near and dear to them who have died of this disease become almost pleasurable. The patience of sufferers under a long-sustained mortal affliction is naturally regarded in the most amiable light. But we must not forget, as psychologists, that comparatively with many of the "ills which flesh is heir to," this complaint occasions less of corporal suffering, and less severely tests the powers of endurance. We should remember this, not that we may cast a shadow of disparagement upon the characters of those who, having had something, probably much to endure, have endured it patiently, and left behind them a pleasing image of tranquil resignation to the will of heaven in the memories of survivors; but that we may be just to such as, having had more to suffer, have naturally, and almost inevitably, displayed more of irritability and impatience. We have noticed how bowel disease induces cerebral disturbance; how, through the medium of physical organization, the mind is made a party in the struggle. We thus see clearly instanced the influence of the body on the mind. The intense agonies attendant upon the passage of gall-stones, or upon a paroxysm of tic-douloureux, are too great to be borne by any human being with tranquillity. We remember having overheard some severe and unkind comments passed upon a clergyman who could not refrain from manifestations of impatience under extreme suffering from the former mentioned cause. It was inferred that he came short of his duty as a Christian minister in not setting his flock a better example. There is no degree of strength of mind which disease and pain may not master. We have thought it necessary in this exordium to our subject to adduce examples of the influence of the body on the mind; some notice of the influences of the mind on the body should also be subjoined. There are the various passions which inspire, exalt, and debase humanity. There are painful or agreeable surrounding circumstances. These take each its part in influencing the health; these constitute some of the links which unite physiology and pathology with psychology. There is also to be considered the influence of one mind upon another, which is great; as likewise its power as exercised within and upon itself, as well as upon the body. There is what is of a loftier order than even intellectual, there is moral power: there is strength of will, of an inferior order to both, but capable of greater ostensible achievements than either. There are none who have not observed the effect of hope as a cordial, of fear as a depressant, upon invalids. Health and longevity depend much upon circumstances, much upon the due management of the mind. A man who upon a sick bed is disturbed by the reflection that he has not succeeded in making a due provision for his family, may be inclined to give himself up to despair, and actually suffer himself to die; or his want of resignation to his fate, his cherished designs for the future, his sanguine determinations to carry out his views upon his recovery, may conduce to his convalescence. The subject is one, not of great only, but of universal interest. To pursue it further here would be to expatiate over far too wide a field. We propose in this paper to segregate for investigation but a small portion of it. We shall devote it, for the most part, to the consideration of the connexion between genital physiology and pathology and psychology. It seemed necessary to adduce, first, a few familiar examples of the connexion generally subsisting between the condition of the body and that of the mind, by way of introduction to the special consideration of one of not the least important links in the chain that unites them. Lady Mary Wortley Montague says, in one of her Letters, "that she had heard speak of many sorts of persons; but that, for her part, she had only met with two sorts—men and women." It is partly upon this hint that we are about to speak, hoping that all due allowances will be made for us as for pioneers who are engaged in breaking up fresh ground. The station we are about to occupy exhibits a *point d'appui* between kindred sciences which has hitherto been seldom, if ever, approached. We shall have to descend from generalities to particulars, some of them sufficiently agreeable objects of regard, but others of them wearing a far from fascinating aspect. We shall, first, enter upon the more agreeable and least laborious department of our duty. We shall find ourselves traversing a pleasant-enough path and in pleasant company, that of poets, sages, and of the sainted among Christian, as well as the renowned among heathen, moralists. We shall have to travel to Milford Haven with Imogen, to thread the mazes of the forest of Ardennes with Rosalind, to gather violets and cowslips and primroses with Perdita. We shall have to follow with Charles Lamb, *haud passibus equis*, at a distance, the steps of Shakespeare; we shall have to follow the steps of a greater even than Shakespeare—of Dame Nature. We may, going higher yet, pursue to its earliest earthly sources, the sacred fount of all inspiration, and undergo ideally with him the seven years' service for love of the Hebrew patriarch. We may go everywhere and anywhere, and find, without seeking, illustrations of our subject. Without looking into the world without, it is much if we cannot most of us look within ourselves with a fair prospect of obtaining considerable information upon the subject. To proceed,—it has been shown how close an affinity subsists between certain physical and psychological phenomena: and, while it has to be conceded that there are diseases which act but slightly and inappreciably as disturbing forces upon the mind, it will be perceived that this concession can least of all be held to apply to organic disease or functional disorder of the generative system; not genital disease indeed only, but the ordinarily fulfilled functions of the reproductive organs while in their normal state much influencing the mind, and producing, as the status of puberty becomes established, absolute and plainly perceptible changes in its character. The same truths admit of being expressed in the rude and uncourtly terms of science, which have given vitality to the poetry of every language. Science, Philosophy, and Song concur in telling the same tale, only that what the latter generalize, Science expounds and specifies: they speak of the master passion, and hymn its eulogy or lament its pangs of discomfiture; Science, of its more gross and corporeal elements.

> "Love lives; Thought dies not: the heart's music still
>   Prolongs its cadences from age to age;
> Perpetuates its melodies, which thrill
>   Through each voluptuous leaf of Nature's page!"

Man perishes; but the passions common to human nature will endure as long as the world exists; hence the interest in them never ceases, never becomes obsolete; hence our sympathy with the joys and sorrows of the long since dead, as if they were yet among the living. The passions, and among these the master passion especially, supply us with countless examples of the agency of the body on the mind, of the mind on the body; of the re-agency of each on the other. With the advance of life, their development becomes more complete, their tendencies and objects more clearly understood. As in the female sex the frame becomes more womanly, a thousand new graces come into view; the mind itself, in becoming more mature, becomes more feminine: in both sexes, the distinctions of sex become more marked and definite. In the male sex, not the aspect and voice only, but the mind undergoes a change. Those changes in persons of both sexes which are of a psychological character are matters of as plain recognition as those which are physical; and so also are any pauses in the march of nature towards perfection of frame, and maturity of mind, which disease or other obstacles and impediments to its progress occasion; and, as we may add also, any forced and unnatural acceleration of its pace. The acclivity from childhood to adolescence may be ascended too rapidly: the ascent itself is not without its perils and difficulties; it extends, indeed, over one of the most dangerous tracts of country which we have to pass in the course of our journey through life. Upon our safe conduct through it, the health of body and vigour of mind of all after life greatly depend. Through educational neglect, there may be hardy, but wild and worthless plants; and a hot-house cultivation may produce such as are only calculated for useless and idle show. There may be, in fact, an extreme cultivation of the mind, which shall tend eventually to incapacitate, rather than strengthen it, causing it to lose in sensitiveness more than it gains in power, prematurely exhausting those energies which are requisite in order to wage the battle of life successfully. During a requisite course of study, habits of abstraction of mind may be formed, which render persons, as members of society, useless, because isolated and unsocial; and which, removing them from the ordinary temptations of man's worldly condition, leave them but a more certain and easy prey to temptations from within.

To the point last named, we shall recur hereafter. We shall proceed, at present, to the period of life's full and favourable maturity. There is a remark made by M. Lallemand, respecting hymeneal excesses, which rather amuses us. He gives it as his opinion, founded upon observation, that such excesses are more frequently committed out of vanity, than from veritable impulses. This may be consonant with French, but we doubt much if it be consonant with English, nature. In the stead of vanity, we should place an inordinate desire to please, of too unselfish a character, to come under the appellation of vanity. But in secular, as in devotional matters, "perfect love casteth out fear;" and the *juste milieu* soon establishes itself. Excess proves its own cure. Persons of opposite sexes, who love each other, soon understand each other. A want of such mutual good understanding implies a want of true attachment. The brain may indeed be peopled with erotic images, which may induce parties to trespass over the limits set by the physical wants of the system; but this is less likely to occur in married life, than during a random career of dissipation. Hence it is that marriage, founded on sincere mutual attachment, is the only condition for the adult of both sexes which is perfectly consonant with nature; in every other state of life, there are complications of adverse feelings, and it is much if there are not irregularities of one description or another; under any circumstances, there are perpetual contests. In athletic sports, in which married are matched against single men, the balance of advantages gained is in favour of the former. More regularity and uniformity of life is, in the case of the former, to be presumed. We think, moreover, that *settledness* of mind (psychologically) must contribute to this physical superiority. There is an escape from the various distractions attendant upon a life of celibacy. Nature does not fail to reward those who act in obedience to her dictates, although her ability to reward it has its limits. Our situations are so diverse, our capabilities and appetences so boundless, that we must not expect in this life to command an assemblage of all the elements whose coalition is required to perfect our happiness. Most of our race have to learn to be content in the absence of many of them. In the attachments, which lead parties to the temple of Hymen, there is more of the psychological, and less of the merely physical, than is imagined, or that might be inferred from reading Lallemand's work. No such thing can reasonably be expected as the most perfect physical adaptation possible. It is quite among the inferior necessities of nature that it should be so. Such adaptation rather succeeds psychologically than pre-exists physically : we are not all flesh and blood, and nothing else. In married life, the *mens divinior* is not without opportunities of displaying itself. There are causes of solicitude arise, which render self-command needful; there are attacks of illness and indisposition; there is a certain period, during gestation, at which abortion occurs more frequently than at other periods, and at which, in many, if not in all cases, the control of erotic impulses is demanded. Many of those kindlier sentiments, which rank far higher in the scale of morality than these animal impulses, are called into requisition. There are none of the virtues which are not required, which are not practicable in married life, and for the practice of which occasions do not continually arise; and for the practice of them, unseen, unknown, not for display, not out of vanity. The most heroic actions of life are those of which no one knows anything, and which are not even suspected to be heroic by the actors of them. Though the mishaps alluded to occur most frequently from the tenth to the twelfth week, weakly female constitutions may, throughout the whole process of gestation and lactation, be taxed too much. At the period named especially, this circumstance should be borne in mind. Mishaps of this nature are, for transcendental as well as utilitarian reasons, much to be deprecated.

To sum up, first, some of those which we venture to characterize as transcendental. A thousand instances, general and special, might be cited, in exemplification of the privations which might have been sustained by our whole species, had many of its brightest ornaments perished immaturely : nor can we surmise what privations we have sustained, and may now be sustaining, through mishaps of this kind, some of which, probably, would not have occurred, had due regard been paid to certain of the laws of nature. Only suppose that the chronicles of such mishaps had included, under the ban of an untimely annihilation, those embryos which, attaining their full growth, would have matured into our Watts and Arkwrights, what changes in the condition of the whole civilized world might have been thus, for many generations, or indefinitely, deferred! How do we know, but that, through any one such mishap, a Shakespeare, a Sappho, a Newton, a Herschel, a Madame de Stael, a Miss Edgeworth, a Mrs. Somerville, a John Hunter, may be crushed in the germ? Setting aside this fear of the untimely demolition of Shakespeares and Sapphos, which is by no means chimerical, there is—THE EVERLASTING SACREDNESS OF HUMAN LIFE, even in its earliest and most rudimental state of being. This is not sufficiently borne in mind in what may be termed the legislative enactments of men against women, in cases of illicit births. It is not right so to enforce chastity as to promote murder. Among the illegitimately born, we have, in comparatively modern annals, Fairfax, the translator of Tasso, the author of one of the most valuable additions to the literature of our country; and in Sacred Writ, Jephthah, one of the heaven-inspired heroes of ancient Israel. Thousands of other instances, both general and special, might be adduced. It is to single individuals, here and there, whom nature has extraordinarily gifted, or who have shown unusual assiduity in studying her laws, that society lies under its greatest obligations. Supposing M. Lallemand to have perished in embryo, the loss to science would have been not the less irreparable for being unknown. There are seasons and circumstances, then, which require that the passions should be kept under due control. However, upon a large scale, all that happens may be said to be right, inasmuch as Providence permits it to happen; we, ourselves, unless this duty be fulfilled, are personally in the wrong. Duties, in order to be fulfilled, must be known: but it is to be feared that, as Sir John Falstaff said to the Lord Chief Justice, we labour less under "the disease of not listening," than "the malady of not marking." There are many things, commonly known, which are not sufficiently borne in mind, even by those to whom they are best known.

Besides the foregoing considerations, there are others, and these such as come more immediately home to us: mishaps of this nature sometimes terminate fatally to the mother; often lay the foundation of life-long debility and suffering; seldom occur without causing some degree of injury to the constitution. There are also, during gestation, various disturbances of the system. There are concomitant inequalities of temper, which require to be humoured, not thwarted. There are sometimes paroxysms of cerebral excitement approaching to insanity, pending which, the most sane procedure is to let the storm blow by. Inconsiderate and ruffianly conduct on the part of the husband causes many of the miscarriages which take place among the lower classes. Children may be killed before they are born, without perishing immediately: they may be born at the due period, and live months, and perhaps years, and yet sink into an early grave through prenatal injuries. There is, however, an obverse to this assertion. It is where the domestic virtues display themselves in the midst of privations, and anxieties, and sufferings that they shine most conspicuously. They are like the snowdrops and crocuses, which unexpectedly peep up out of the frost-bound soil, to diversify the depth and dreariness of winter, and give us a cheerful foretaste of the coming spring. While among those whose whole existence is a toil and a martyrdom are to be witnessed proofs of daily self-denial, we meet, too frequently, among those who have all the conveniences of life at command, persons incapable of the small self-sacrifices which common humanity dictates. It is not perceived that acts of self-denial which promote the happiness of others, reflect back happiness upon ourselves. There are allowances also to be made by, as well as for, the gentler sex. Upon the whole, marriage, with all its alleged asperities, taking it as it is, rough and smooth, does not require to be looked at through the medium of a Claude Lorraine glass, but regarded in its true light, as less a matter of appetites gratified than of affections and attachments perpetuated, and requires no finery of rhetoric to deck it out.

We have wished to make it out that self-denial is Christianity carried out into practice; that it is the perfection of MORAL SANITY; that it conduces to, that it is, happiness. Of all deviations from this golden rule, Guilt and Insanity share between them the odium. To affairs of the heart and passions we have sought to apply this axiom, no less applicable to these than to all others. M. Lallemand's work is a remarkably faithful history of certain human instincts: it does not go beyond, nor profess to go beyond, this: it is in so far, therefore, ethically imperfect. There is another view of the question of abortion which we have not omitted to place before the reader out of forgetfulness, but because it does not at all alter the case. It may be said that, as a set-off against a like number of extinguished bards, patriots, sages, heroes, heroines, and savants, are among those who perish in embryo, some who would perhaps have lived to expiate heinous offences against society upon the scaffold. With this we have nothing to do; of this we are not constituted judges: we are not enlightened as prophets with any inspired knowledge of individual human destinies; there is one thing of which we are well assured—the inalienable sacredness of human life. It is owing to our sense of this, so far as we possess it—and we possess it comparatively speaking in a high degree—that we have been saved from the internecine carnage which has desolated many of the principal continental cities. The very cowardice of the disaffected among us does their hearts inexpressible credit; in a pure and good cause, Englishmen are always brave; it is only in a bad or doubtful cause that they are pusillanimous. The trammels of sound religious and moral education are not easily thrown off. To resume our subject, and before quitting it to enter more closely into some particulars hitherto slightly touched upon. The physical congenialities and discrepancies of connubial life form a most fertile theme for discussion: nor is it blameable, but on the contrary highly meritorious, to seek, upon this as upon every important subject, "to add to virtue knowledge." Congenialities, in some respects, may co-exist with discrepancies in others. Wherever science turns her calm and unimpassioned eye, she discerns much which, by the untutored in her mysteries, passes by unrecognised and unmarked. Not to adduce any abstruse and far-fetched example of this, let us be permitted to unfold a little family picture, sketched, not from imagination, but from real life. There is a mother, surrounded by a young family, sitting in a child's chair nursing her infant. What is observable in this? That she has a pelvis of much less than ordinary capacity. Her husband is just entering the room. You stay awhile. You are about to take your leave. Lay hold of his hat under pretence of mistaking it for your own, and attempt to put it on. You find it barely perches on the tip of your head. Had it been of a size so large as to have flapped on to your nose, in other words, had he not had a below-average sized head, she would, more probably than not, have died in her first accouchement. There is such a thing as Providence. It need not be said that no such thought as that of the size and configuration of her suitor's head ever entered into her mind during courtship. It will be seen also that there are circumstances under which sterility, in so far as life is of value, would be a fortunate incident. We perceive also in the foregoing sketch the occasional advantage, quantitatively speaking, of want of brains. We cannot expect in marriage the most perfect possible physical adaptation in this or in an erotic acceptation of the term. Where this is the case in the latter sense of the term, a marriage may, nevertheless, be without offspring. We may easily conceive how a man may wed perfect physical adaptability, all the talents, and yet have for his wife a Messalina or a Lucretia Borgia; or, on the other hand, all the virtues and graces, without the qualities which physically characterised the more beautiful and less precise of the goddesses of ancient fable. The wheels within wheels of human fate are complex and countless. We have said that nuptial happiness is more dependent on psychological, than on physical qualities. It is certain, however this happens, that incongeniality of taste upon matters of no moment whatever disposes persons to hate each other more inveterately than really important discrepancies. There is a very pretty semi-platonic theory prevalent which makes out mental and moral congeniality of sentiment to be requisite to the phenomena of reproduction—a theory which displays a failing to which all theory is much addicted—that of being false and unfounded. Nature does not leave the consummation of this object to the incertitude of existing or pervading mental emotion. It is a pretty theory enough; but like causes produce like effects: whether parties hate, love, or are indifferent to each other, nature knows no variation from the fixed laws which govern her proceedings; statistics show the same average results. Shakespeare says :—

> "There is a Providence that shapes our ends,
> Rough-hew them how we may."

There would be less care corroding human hearts upon these matters, were there a clearer conception of how nearly upon an equality are all human conditions in so far as they depend upon natural causes. The want of nothing, the attainment of which is placed out of our own control, should be suffered to render us unhappy. Apparent benefits withheld may be in effect curses from which we are shielded. Such a state of mind confers happiness, displays true sanity. We little know from what unsuspected evils the most unlikely second causes may have preserved us; as little as a family driven past a precipice at night by a drunken coachman know how often and how narrowly they may have escaped destruction, and this perhaps owing to the drunkenness of the coachman, and his bold driving under circumstances which, with a timid driver, would have proved fatal. Prometheus is said, by Æschylus, to have conferred a great boon upon mankind in sending "blind and hoodwinked hopes to dwell among them." What we speak of is a something much better. On matters wherein reason and volition avail nothing, there is still left to us that lively faith in the guardianship of Providence which will carry us through every contingency, and the absence of which constitutes the truest unhappiness. Persons driven about by every wind of doctrine, at the mercy of every variation of circumstances, have never been perfectly sane; they are dwellers all their lives in the very vestibule of insanity. Where true sanity of mind has not been fixedly established on a sound moral basis, its overthrow is easy. When we speak of deformity, we must have formed in our imaginations some model of real or ideal beauty; so to understand insanity, we must know what sanity is. We have to take cognizance of variations from some standard of perfection. Accordingly, we have thus far dwelt chiefly upon the normal in physical development as accompanied by intellectual sanity and moral correctness of conduct, and soundness of principle. It is henceforward our less pleasant task to visit the asylum and "the lazarhouse." We have to quit physical perfection and mental sanity for their sad reverse. We do not forget that from a tenth to a twelfth part of the number of insane females owe to the reproductive organs the origin of their insanity, whether during gestation, parturition, or lactation; but these subjects have been treated in a former number. We shall but add here our impression that cases have often been treated, and successfully too, as phrenitis, which have been in reality cases of metritis, but for which a treatment more lenient and more local would have better availed and at less cost to the constitution. Leeches have been applied to the temples, which ought to have been applied to the abdomen, the cerebral disturbance being only secondary and symptomatic. On the other hand, it has sometimes been suffered to become fixed, to assume the position of primary disease, to remain irremediable, from want of a well-timed and placed application of leeches. There is usually a period at which the brain is to be relieved by subduing, not cerebral, but uterine inflammation. We put forth this suggestion as the result of much experience. It is a case in point that the seat of puerperal insanity is the uterus, not brain.

It is said that "*nemo repente fuit turpissimus.*" Following this rule, we shall proceed "from bad to worse" by gradations. There is one cause of continuous gloom; of a repining which does not amount to insanity, but often amounts to unhappiness; connected with the reproductive organs. It may be summed up in the complaint of Macbeth against the witches—"that they had placed a barren sceptre in" his "gripe." We have touched upon this already; but there is more to be said of it. It is possible to treat this subject cheerfully, and cheerfully we shall treat it. One design of Providence in the institution of marriage is, doubtless, the perpetuation of our species. But as individuals we design no such thing: our purpose in the main is to make ourselves happy; and if we are not so, it is our own fault. There appears to be no prevalent apprehension lest the designs of Providence should upon the large scale be frustrated; nor does any one marry out of an impression that the queen is distressed for want of subjects. There are sometimes, however, both among the single and married, sensations which hurry parties to, and sometimes over, the verge of sanity. There are impressions which, *exceptis excipiendis,* and the exceptions are very rare, are entirely unfounded. The whole subject is ill understood; requires to be better understood. The horrors of mind attendant upon a state of imaginary deficiency, are owing partly and considerably to the stimulus of continued orchial secretion greater than those attendant upon real incapacity. That there are such horrors at all is tolerably good presumptive evidence that they are gratuitously entertained. Where there is an absolute deficiency of genital power,—where, in other words, incapacity is real and complete, especially where it is congenital, there are no such horrors. The party commonly becomes both a *gourmand* and a *gourmet:* there is plenty of adipose secreted and deposited: their *embonpoint* testifies to their tranquillity.

There is almost room to doubt whether the most perfect forms of cerebral organization are easily transmissible, especially in the male line. We only remember in this country one conspicuous example of the reverse of this difficulty or incapacity of transmission in the history of the Sheridans, who, generation after generation, from the friend and crony of Dean Swift, down to the Honourable Mrs. Norton, have succeeded to the heritage of extraordinary talents. Against their genealogy and that of a family no less celebrated in science, the Herschels, might be brought a numerous and striking array of obverse examples. Who ever heard of any descendants of Swift, Pope, Johnson, Collins, Goldsmith, Savage, Tasso, Petrarch, Rousseau, De Stael, not to name countless other eminent authors, except their writings? The only descendants of Shakspeare, Milton, Byron, were daughters; Milton's line becoming extinct with the death of his grand-daughter: the only son of Napoleon, the only son of Sir Walter Scott, died young: as if where extraordinary cerebral organisation were in the male line transmissible, longevity should be denied it. Indeed, where there are descendants, it is commonly property and a name that are inherited, not talents. Should any persons sigh for the possession of genius by their children, the records of the misfortunes of men of genius may well console them. There are nosologists who have, in fact, spoken of genius as being a disease of the scrofulous kind. Had Pope, Johnson, Rousseau, and many other great men, been born in China, their parents would have destroyed them as not worth rearing. What the metaphysical gains, the physical loses; and the reverse: both seldom coexist in perfection. We question whether it is too much to say that the man who is a clown with a large family of children might, otherwise educated and circumstanced, have been a poet or philosopher with none. In Burns we have an example of the *physique* and *spirituel* in a remarkable degree blended: his writings, now sublime, now homely, attest this combination. Like Sheridan, he was more a *real life* man than most of our poets. Shakespeare, indeed, was everything; but he is too high above all common height for speculation. Upon the whole, it would seem as if we should almost consider a man's having a large family of children as circumstantial evidence of an inferiority of cerebral organisation rather than the reverse. Genital incapacity itself, however utter, under this view, and if, indeed, it be true that the mind is more than the body, should less be regarded as a misfortune, than as a proof that God distributes his good gifts to men equitably, refusing to some common gifts to whom he has imparted such as are extraordinary : and, where he has not imparted such, providing otherwise here or hereafter indemnifications for all privations and ills sustained. It is in accordance with the same system of equity that fame and wealth seldom fall to the lot of one and the same individual. Men are sometimes so placed as that they must consciously and knowingly forego the acquisition of one or the other, the means for acquiring each being different, and the possession of both unattainable.

If the maxim of "*nullum magnum ingenium sine dementiâ*" be correct, the "dementia" of itself constitutes a sad drawback; and there is, doubtless, too much of truth in it. How often and how far this "dementia" is associated corporeally with the reproductive organs, the lives of Tasso, of Sappho, of Petrarch, of Rousseau, of Byron, of Burns, of hosts of other poets, too painfully illustrate. The unfortunate amours of many men of genius have, unavailingly to their own personal happiness, associated immortality with human passion. "They learnt in suffering what they taught in song." Ambitions misplaced, unrequited attachments, formed the burden of their most sublime and sweetest minstrelsy. With these the songs of the troubadours are fraught to overflowing. So is all song; from the Canticle of Solomon down to the song of Jeannette and Jeannot, which we daily hear carolled in the street,—all verse tells the same tale of love; a tale which never wearies, when well and truly told. And why? Because it tells of hopes, fears, solicitudes, common to the whole human family. These, poetry not describes only, but etherealizes and exalts.

> " There, in that gift of God, Imagination,
> Is a true nobleness beyond all pomp!"

To these hopes, fears, anxieties, we revolt from applying the terms of science, however strictly applicable. We will not, just here and now, beckon to them to descend from their pedestal : we will there, eloquent in their silence, leave them, grouped and draperied witnesses to the truthfulness of our assertions. Science shall stand veiled, as best becomes her, in their presence.

But the "*facilis descensus Averni*" is before us: we must quit them and descend : it shall be with reluctantly averted eyes and by gentle steps. The golden rod is plucked; we proceed; we see the dark shades of Erebus flitting about us. Our severest toils are yet to come. From the monuments of the loves of the poets, from the tomb of Abelard and Heloise, we turn away, not seeming to go, with the purpose of Æneas, to seek oracular advice of the disembodied, but rather in the spirit of Orpheus for the rescue of Eurydice. But our path, as it grows more gloomy, reminds us of the real intent of our expedition. We leave the physically healthful, the psychologically sane : we perceive ourselves entering upon the dreary regions of disease and insanity; we pass them; we find the shadow of death around us: all due rites having been performed, we seek—we summon forward the shade of Jean Jacques Rosseau. We go not alone, but with Lallemand and H. J. McDougall, his able translator, for our conductors, who introduce us to him. It is not the substance of the communications made, but the results of the consultation of this high oracle that we shall give.

The interview, be it then said here, was of a nature rather to exalt than to disparage the character of Jean Jacques; if not to rescue, to raise it. His eloquence was the inspiration of disease; but disease never before or since, unless it be in the case of our own Cowper, spoke with a voice so eloquent. There is reason to believe that his moral aberrations owed their origin to a congenital cause; that had Lallemand attended him, or had the point in one department of surgical science been reached to which Lallemand has advanced it, his disease might have been cured, and his moral and intellectual sanity thus established. The powers of his extraordinary mind would not, in this case, have been frittered away in portraying the symptoms of an, at that time, incurable complaint. Happy himself, his writings would not have so tended to the world-wide dissemination of political and social discontent. The tree which disease so fatally ingrafted would have borne less bitter fruit. A half-farthing's worth of nitrate of silver skilfully applied, and France might have been spared many of the horrors of her last century's revolution. As it is, it would perhaps have been better for his country and his species, had his abilities been petted and pensioned into nihility. Mighty effects often depend upon insignificant causes. Why is not Ireland at this moment a Protestant country? Because Oliver Cromwell's doctors were afraid of giving him a few doses of Peruvian bark. He died of an intermittent fever, of which, in all likelihood, quinine would have cured him. Had he lived, he would not have stopped at less than the entire subjugation of that island, the coasts of which have been so strewn ever since with wrecked administrations, and in whose sisterhood our own island experiences much such comfort as might be derived from living in the neighbourhood of a volcano. A more entire amalgamation, however conduced, resembling that of old between Saxon and Norman, would have certainly tended, after the lapse of nearly two centuries, to the promotion of more good fellowship than subsists at present. We only cite this as an additional instance of the dependence of great effects on slight causes: as such it is well in point.

We have spoken of the reproductive organs in relation hitherto to their use, as opposed to their abuse. Rousseau speaks of a certain vice, which he confesses as having "charms" for a "vivid imagination;" and elsewhere as "a means of preserving his purity." He does not mean, by the latter phrase, his moral purity; he does not say anything so depraved and hypocritical; he means his preservation from physical contamination. Lallemand shows that Rousseau's was an actual, not imaginary malady; that disease caused, or fostered, or instigated, in much or little, the moral aberration alluded to: that the symptoms were those of spermatorrhœa. In Mr. M'Dougall's able translation of Lallemand's work, the chapter relating to Rousseau is not introduced. M. Lallemand, in some passages of his work, supplies us with a glass, which almost throws a *couleur de rose* over self-abuse. He asserts (in page 235 of Mr. M'Dougall's translation, to which we refer as most current in this country,) that there are many, who, solely through their being more continent than virtuous, plume themselves on having never indulged in either natural intercourse or self-abuse, and who ascribe to strict views of morality and religion an exemplary course of life, which really only indicates a want of genital power. We participate in his detestation of hypocrisy where there *is* hypocrisy; we acknowledge his clear-sightedness in piercing through the mists of self-illusion; we admit that all phenomena induced by spermatic plethora show genital power; but we think his opinions require to be qualified, and, by being expressed somewhat more guardedly, placed more out of the way of misinterpretation. It is left too open to the reader to doubt whether there be such virtues extant as those we have been in the habit of calling "temperance, soberness, and chastity." There are frequent instances in this country of a healthful frigidity of temperament, arising partly from nature, but chiefly from the moral restraint imposed upon the passions by a strictly religious and moral education, which it takes time and the sunshine of a true attachment to thaw; but which, thawed, gives place to a warmer and more lasting summer than succeeds to many a more lavish spring. In minds of much polish, the intermediate steps between the love of sentiment and the love of passion must be many. Fidelity and permanence of attachment are not to be expected from those whose passions have blazed forth too early, and too vividly and diffusely; who approach the altar of Hymen with impaired hearts, if not impaired constitutions, in a dissipated and dissolute frame of mind; who, having practised excess, do not easily, if at all, settle down into the tranquillity of domestic happiness. Vagrant desires have been excited, which, till those they have agitated take their everlasting rest in the bosom of their mother earth, will never know repose. There is the "hardening of all the feelings," of which Burns sings; "the madness of the heart," which Byron, in so many passages of his writings, develops rather than portrays. There are no deviations into error which are irretraceable; but we speak of what is common. We have taken upon ourselves to view the subject ethically as well as physically; ever purposing to adhere to truth; to such adhesion to sacrifice everything.

Spermatorrhœa, of which hy pochondriasis and monomania are usual symptoms, does not always arise from self-abuse; it has other causes, which we shall specify hereafter. Unlike many other complaints, it never spontaneously cures itself. Till it be cured, none of its symptoms, physical, moral, or psychological, cease. There are few cases which, other means failing, are not curable by cauterization. There is thus one species of insanity associated with the reproductive organs which surgery can absolutely cure. The existence of spermatorrhœa is known by the presence of spermatozoa in the urine; by diurnal pollutions, which take place more or less passively; and by the size, number, and appearance of the spermatozoa which microscopical examination discovers in these secretions. Spermatozoa, as they exist in healthy spermatic secretion, have been made objects of microscopical examination almost from time immemorial; they were in old times called homunculi, and were considered to be little men and women in a tadpole state. We do not think M. Lallemand's opinions of their nature and functions much more feasible. He conjectures that they assist the orchial secretion on its way towards its procreative destination. We think that the peristaltic action of the intestines might almost as well be ascribed to the stir and bustle of the infusoria contained in the fluids we swallow. We accept of the differences in size, shape, number, and development of the spermatozoa found in healthy and in depraved orchial and nephritic secretions as, symptomatically speaking, conclusive; but are inclined to think that these differences are owing to alterations that have taken place in this fluid as being the element in which they are generated, and in which they can only be developed perfectly and numerously when it is in a natural and healthy state. We regard them simply as a species of infusoria, whose condition and appearance betray the poverty or richness of the soil of the territory they occupy, and the circumstances of their birth and breeding. When the orchial secretion has become, in an extreme degree, depraved and watery, the spermatozoa are few in number, imperfectly developed, more diaphanous, less easily detected; they are found scattered about here and there, like the gamy and high-flavoured and half-starved sheep that wander over the wilder regions of Scotland, or like fish in rivers rendered untenantable by receiving the contents of common sewers or the refuse of dyehouses. These spermatozoa, then, are either homunculi; or infusoria, whose presence is incidental and of no physiological importance, or as accessories to the phenomena of conception. We are not disposed to adopt the *media via* of M. Lallemand. We think that it is the quality of the fluid that is essential, not the state of health of its inhabitants. It is a question of probabilities. As, what they were once supposed to be, homunculi, no doubt an ingenious line of defence might be set up in their favour. As a question of no practical importance, and one which, if it were, it would not be very easy to settle, we might very well leave it open. Their pathological value, as indications of the state of the orchial secretion, is indisputable. M. Lallemand's discoveries constitute quite an era in this department of medicine and surgery.

Having fairly broken the ice, we deem it right to pursue this least agreeable, but most practically important portion of our subject, leisurely and systematically. It appears to us to have been treated amiss, because only physically, and not also psychologically, investigated. For instance, among the symptoms of spermatorrhœa as revealed by Rousseau, and by others of less eminence, but rivals to him in suffering, are two which are of a strictly psychological character,—the fear of hell, and the inability to meet others eye to eye,—both symptoms of either insanity *or* a bad conscience. Supposing the individuals to be sane, there is no concealing that they are indications of mental or moral distortion. Its physical phenomena have been nowhere treated so ably as in the work of M. Lallemand, which is not an *ad captandum,* but a truly scientific production; and one which, although we do not consider all the author's conclusions to be conclusive, is evidently written in perfect good faith. To say this is to say much of any work, especially of a work on such a subject. He has not only thrown much light upon it, but, more than this, is almost, if not quite, the discoverer of a hitherto unsuspected disease; and, better still, of an absolute cure for it in many, of the most appropriate remedial treatment of it in all, or in most cases. But the more merit a work has, the more necessary is it to point out any errors that may be detected in it. We shall return to the notice of these as occasion offers, adding only here our sense of the diligence, care, and patience which he has displayed in the course of his investigations, and his caution in what might be termed summing up evidence. With regard to all works upon specific diseases, which are rather addressed to the non-medical public than to the profession, suspicions of their object are apt to be entertained: they sometimes meet with the misfortune of being looked upon in the light of advertisements, especially when they treat of maladies with respect to which the imaginations of patients are peculiarly accessible. No one can by any possibility place under this category the work of M. Lallemand, or Mr. McDougall's translation of it. As before observed, we do not coincide in all his inferences, and wherein and why we do not shall be in the course of this paper explained; but the main fault which pervades his work is, what some will consider rather an excellence than a defect, its not being sufficiently psychological; or, to speak more accurately, its not being sufficiently *ethical.* We have taken the liberty of speaking of temptations from within. Allied with these is one infirmity or vice of both childhood and adolescence, which, whether it be pronounced immorality or insanity, as the evidence of the case may be, tends to a termination in the latter. We shall exert our utmost energies to arrive at and promulgate as much as can be attained of the exact truth concerning it. Every public misrepresentation, however it may be put forth out of some sickly, and fantastical, and dwarfish notions of doing good, is a serious offence against the community at large. The influence of mind upon mind is capable of, and is liable to, much misuse. The power which a medical man wields over the reason and imagination of his patients requires to be employed with extreme circumspection; such power is sometimes exercised, when not dishonourably, indiscreetly. Medical men who put forth opinions *ex cathedrâ,* should mind well what they say; it has sometimes happened that, like Frankenstein, they have evoked into being a monster whom they would afterwards fain annihilate, and who causes them much annoyance and uneasiness, but of whom they cannot at option rid themselves. The word has gone forth; " a carriage and six horses could not fetch it back again;" and to the minds of the susceptible " words are things." Mischief may be soon effected, which it is found difficult or impossible to repair. If, on the one hand, truth should not always be told to patients, we think, on the other hand, that falsehoods should never be told them. In deliberately written medical compilations the more especially, there is no excuse whatever for one hair's breadth deviation from the line of honesty. We may err through want of knowledge, of evidence, or want of reasoning powers, or through a want of sufficient application and patient research; but honesty consists in saying what we know and think; in asserting nothing we do not know as if it were known; and in not professing fixed articles of belief on subjects upon which our mind is not made up; in stating our formed opinions, and the basis on which they are founded, candidly; in leaving still fairly open all that appears inconclusive. In M. Lallemand's work, unlike those of some who have followed in his wake, the cases are carefully drawn up; they display much shrewdness of observation; much industry of research; there are no rash inferences drawn; there may be some that are incorrect, there are none that are rash. We have in abundance compilations consisting half of common-place truisms, half of a tissue of dangerous plausibilities; containing nothing original that is not crude and unattached and unintelligible ; nothing borrowed that is not copied distortedly and inaccurately; but M. Lallemand is a genuine discoverer in science, a pioneer through regions hitherto unexplored: and to discoverers in science, as to creators in literature, may the higher honours which are their due be ever paid cheerfully. Science, like Genius, knows no country. For us, we shall acquit ourselves in the humble office we undertake quite to our own satisfaction if we can place the subject in true perspective before the view of the reader, upon the reduced scale which our limits necessarily prescribe to us. So far as relates to that department of pathology of which he treats, we shall gratefully accept of M. Lallemand's assistance and authority. We shall not, however, limit our remarks to physical symptoms exclusively. In order to make the subject more clear, we shall precede a close and particular synopsis of it by advancing certain general propositions.

Violations of physical laws meet with physical—of moral laws with moral—of psychological laws with psychological, retribution: such is the rule—if there be any rule which has no exceptions, it is this. Persons who pilfer are not liable to have whitlows form on their fingers as a penal consequence of pilfering. If a man sustains bodily injury from a tile blown down upon him from the roof of a house, the accident causes him no sensations of remorse. It causes a man neither bodily suffering nor any of the pangs incident to a guilty conscience to be beaten at chess. He may violate all three codes of law at once in one act, in which case he will be visited by all three with retribution, as would happen to him, if in the course of perpetrating a burglary in an unskilful manner he should get knocked on the head. But there is in none of these examples any confusion of relationship between causes and effects. In the case of the summarily punished housebreaker, who has displayed a want of skill in an unlawful avocation, there is experienced, in return for his violation of intellectual laws, a sense of vexation at his own clumsiness and consequent ill success; and in repayment of his violation of moral laws, he finds the moral sense of the rights of property entertained by others roused up against him to his personal injury, and has probably to undergo the reproaches of his own conscience as well as the dread of farther retribution to come. His errors (so to speak) being complex, his punishment is complex also. In the case of the solitary vice which we are about to bring into question, it is incumbent on us to discriminate between the phenomena observed, and to classify them accordingly, as being physically, morally, or psychologically retributive. The thief who is knocked down in the act of thieving; suffers more or less physically in proportion as the injury sustained is slight or serious. So in syphilis or gonorrhœa, physical suffering bears no proportion whatever to the degree of moral delinquency: it is a matter of temperament and constitution, and of the quality of the virus to which certain parts are subjected. Distinctions must then be made, as before observed, between such consequences of the malpractice in question as are physical, such as are ethical, such as are psychological. The thief is worse punished than by whitlows on his fingers by an accusing conscience, and, if taken and convicted, by the penal laws of his country. The man who should deny that pilfering is punished by whitlows on the fingers is not on this account to be held up as a person who advocates theft. On the contrary, if it were not punished at all in any known way, it would be right to speak of it as a crime which may be committed, secularly speaking, with impunity: it is always right to speak truth. Wrong doing would nevertheless still be wrong doing; crime still crime. We should never condescend to say anything that is disingenuous. In treating this subject, as every other, we should proceed in the spirit of calm research, desirous only to arrive at the exact truth.

Solitary emissiones spermaticæ may occur,—

I. Involuntarily. "When it occurs spontaneously during sleep, in a healthy and continent individual, it doubtless," says Lallemand, "exerts a beneficial influence on the economy, by freeing it from a source of excitement, the prolonged accumulation of which might derange the animal functions." "In these cases," he adds, "it has an effect analogous to that produced by the epistaxis, common and beneficial during youth." (We cite M. Lallemand, without fully concurring with him on this point.)

II. Voluntarily. When such crisis of accumulated secretion is anticipated under the excitement of erotic reveries, in place of erotic dreams. (We think, the foregoing postulate being conceded, this follows.)

III. Voluntarily. When the habit of yielding to the growing influence of this infatuation is established.

IV. Involuntarily. In those cases in which spermatorrhœa, owing to whatever cause or causes, exists; in which emissiones spermaticæ seldom take place actively, often wholly passively.

Not to speak of extreme cases, such as that of Richerand's shepherd, and to avoid speaking of such, not because they are extreme, but because they are rare, if self-abuse causes, physically, a certain set of symptoms, the question is, in what way does it so act as to cause them? It must act appreciably and discoverably. Is it to the amount of constitutional excitement, as excitement, or of the orchial secretion as such, and with relation simply to the quantity of it, that the alleged mischief is to be ascribed? Or is it wholly qualitative, as being self-induced? Taking the question first as simply quantitative, it comes precisely under the same rule as excess in coitu naturali, differing from this only qualitatively. We are warranted in placing them, quantitatively, on the same footing. What may be deficiency or privation, what moderation, what excess, in the latter case, is a matter of individual experience, scarcely admitting of any limit or prescription. What would be excess in one person's case, would not be so in another's. That only is excess which is proved to be excess by a person's health suffering from it. That is not excess concomitantly with which a state of generally good health is maintained. All that we say, quantitatively speaking, of excess in coitu naturali applies equally to self-abuse, if the amount of orchial discharge be alone considered. We mention this the more pointedly, because we well recollect having met with books, written with good intentions, their object being to deter young people from the practice of this vice, wherein calculations are made showing how each emissio spermatica causes an outlay of the *vis vitæ* equivalent to the loss of an incredible number of ounces or pounds of blood. Excess is excess, and we cannot commit excesses with impunity. Morality never requires to be crutched upon falsehoods: in the foregoing assertion there is a sad want of verity: after established puberty, nature provides for the practicability of coitus interconnubialis for an uninterrupted continuance, so long as life, or at least so long as vigour, lasts, without subjecting the party or parties to a tax upon their constitutions by any means so heavy as would be imposed upon them by a like series of abstractions of blood by means of venesection. The question, then, is clearly not quantitative any farther than as the former question is quantitative. Taking into view any given continuous decade of married life, there is nothing probably in which nature is a better guide. But having settled the question of quantity, there is quality also; there is the mode in which the emissio spermatica is induced; we have to consider it as self-induced. There is an abandonment of nature; and Nature ceases to smile on, and to guide, those who forsake her paths. The question of quantity, indeed, is only settled as a question *per se:* we have yet to consider how far this is qualitatively determined. We beg our readers, moreover, to bear in mind that we are at present treating the subject in strict relation to its physical, not its moral, features. It is our part to proceed some steps farther, divested of all moral preconceptions whatever. In coitus naturalis, there are two wills to be consulted; in this vice, one only. In the latter, there may be a want of inducement, there may be the want of a check: it is the want of a check that is most usual: there is therefore more likelihood of excess both of sensorial excitement, and of emissio seminalis to a degree which the constitution will complain of, and nature will resent as extortionate, in the course of a practice of this vice, than in the course of a practice of concubitation. But this only amounts to a likelihood: there may be such excess in either as to injure the health: self-abuse may make such approaches to the involuntary and unconscious, may take place so infrequently, as not to affect the health, except salutarily. It is when it becomes, like opium-eating, an established infatuation, that any dreadful physical retribution ensues. The retribution then is sometimes most severe: the practice of this vice innately tends so to establish itself. There are fortunately, however, many checks to its establishment, which sometimes avail, and sometimes do not avail. We are to do what is right, not what is, or is fancied to be, salutary. But that is a moral consideration with which we shall have to do hereafter. We are now treating it exclusively as a matter of investigation in physics, not as a theme of ethical inquiry. Quitting the path of *à priori* reasoning, we shall find that this vice has its memoirs, its history, its biographies. There are cases which can scarcely be said to be cases at all; there are average cases; there are extreme cases. As in syphilis and blenorrhagia, the physical punishment bears no proportion whatever to the degree of moral delinquency. The natural history of average, or to speak more correctly, of usual, cases will be much as follows. At the age of puberty, persons, from tradition, books, conversation, experience, nature, obtain a knowledge of certain circumstances connected with their physical condition that was unpossessed by them before. A species of sixth sense is awakened into being. There are certain changes in the physical condition. It is not an uncommon thing for boys at school, at the initiatory period of such changes, to teach each other to perform certain experiments in natural philosophy upon their persons; to adduce and to boast of certain proofs of puberty. In other instances, such experiments are practised spontaneously and solitarily. We have to do now, and while proceeding on this especial line of investigation, with what *is* and *is not*, not with what is or is not right. There is sometimes a course of evil habit established; it goes sometimes to great lengths; but this, as we are disposed to think, but seldom: in the case of Richerand's shepherd, with which every medical man is familiar, there was an uninformed mind, there was a singularly secluse avocation. But in the usual course of time and life, there come shame and enlightenment of mind. There are interruptions to such habits. There is the devotion of the physical and mental energies to various pursuits which engross those energies. Those energies are not unlimited; and, while healthily employed, vice itself necessarily fails to monopolize them. Society finds a thousand modes for their employment and expenditure, all of which act as safety-valves to the system, both physically and morally. From the cabin-boy, who is kept upon the run on board ship, and whose indolence and negligence, if indolent and negligent, are followed up by the application of a rope's-end, to the man reading hard for honours at Oxford or Cambridge, the various duties of various stations in life exact the devotion of the bodily and mental powers. These powers have their limits; and their exercise in the path of duty renders excess in the vice alluded to improbable, if not impracticable.

Attachments to woman and to virtue will be formed. It is a complaint which, for the most part, effects its own cure. The degrees in which it exists are various. There are beyond question cases in which, physically speaking, it is severely visited. There is lassitude of body, incapacity of mind; there is frequent micturition; there are disturbances of the genito-urinary organs: there is sometimes spermatorrhœa with the thousand ills attending in its train. In cases less extreme, there are self-reproaches; there is an accessibility to the frauds of designing empirics, who lead their victims many a painful dance through the thorny labyrinths constructed for their entanglement. They have to pass through hallucinations of terror and misery, even if they remain physically unscathed. The physical ill consequences of this vice, except in unusual cases, are trifling; but there are not wanting those who, for the sake of gain, are willing to make much of them. Spermatorrhœa is produced by many other causes besides this, which does not invariably, nor yet frequently, but only occasionally, terminate in spermatorrhœa. By whatever cause induced, it admits of treatment, and generally of cure. One important physiological fact is confirmed and illustrated by M. Lallemand—the facility with which disorder and disease are propagated along its mucous lining, throughout the genito-urinary system. The return of spermatorrhœa upon the retrocession of cutaneous disease comes rather under a general rule than a special regulation. The sympathy of the skin with the mucous membranes is more than sympathy: the skin being continuous, although not identical, with the internal mucous membrane, the cuticle of the former being merely its indurated exterior. This species of metastasis is not infrequent to whatever disease of any internal organ a patient may be pre-disposed. When tinea in infants is cured too speedily by external applications, without a course of alterative medicine, such metastases often prove fatal. Nevertheless, we do not think that, in that section in which cutaneous disease is treated of as one of the causes of spermatorrhœa, the conclusions of M. Lallemand are quite correct. We should rather pronounce, perhaps, that his opinions are in that section expressed somewhat unguardedly. In some of the cases he records, the patients have been worn out by a complication of diseases of which spermatorrhœa is rather the assumed than the clearly detected origin. There is one source of error in relation to the discovery of spermatozoa in the urine that should be borne in mind. Emissio spermatica does not occur in the manner of an arrow discharged from a bow, but by a succession of jets as from a fountain. There may be the presence of spermatozoa in the urine owing to this circumstance and to the desire for micturition which is apt to be experienced after each emissio seminalis. Their presence may in some cases only prove the frequency of this, however induced. M.

Lallemand quotes and confirms the ancient medical adage, "*raro mingitur castus:*" it might also be said *rarius mingit castus.* The affinity between all the genito-urinary organs has been well made out by M. Lallemand. Practically, the application of nitrate of silver in the chronic stage of blenorrhagia and in gleet, as in spermatorrhœa, would seem worthy of careful trial. We have occasion to remark throughout the work of M. Lallemand how much depends upon judicious discrimination—how much danger attends upon the treatment of cases empirically. M. Lallemand does not treat all cases of ascertained spermatorrhœa by the application of nitrate of silver, without reference to its causes, or the conditions which the case presents. We recommend to our medical readers a careful perusal of his work, or of Mr. McDougall's excellent translation. Before quitting this department of the subject—namely the strictly physical ill-consequences of masturbation—we wish to repeat, that it does not, as the rule, cause spermatorrhœa, which only results occasionally from this cause, and then, for the most part, when it exists conjointly with other causes, and that it is a complaint which is also often seen as the result of other causes uncoupled and unconnected with this. There are those who find and make it to their interest to represent this otherwise. When it causes spermatorrhœa, it of course may be said also to cause all the symptoms which characterize this complaint. To John Hunter's remark, that apparatus designed for a twofold purpose does not act so well as apparatus devoted to one special purpose, we might have added one self-evident assertion of Lallemand, to the effect that, among animals, quadrupeds show that they would commit acts of abuse if they could, but they cannot; monkeys can and do. Comparative physiology has its points of interest as well as comparative anatomy.

We next come to those results of this species of moral obliquity which are psychological rather than physical. We much suspect that there are few of either sex in whom erotic reveries have never at any time anticipated those healthy and continent *pertes seminales involontaires,* which Lallemand speaks of as the natural results of erotic dreams. There is no doubt that what he speaks of may and does occasionally occur; but we do not accept without demur of his interpretation of this circumstance as a consequence and a proof of either continence or health. *Tout au contraire,* we should deem it a proof of some degree of moral weakness conjoined with an equivalent of physical debility. "*Les desirs toujours reprimées cessent renaitre.*" There is no periodicity in paroxysms of erotic excitement among human beings as among the inferior animals, nor other periodicity, except such as we ourselves may have established into habit. If it be worth while to investigate any subject at all, it is worth while to investigate it thoroughly. The *pertes*

*seminales involontaires* of which Lallemand speaks as continent and healthful, demonstrate neither perfect chastity nor perfect genital health. But, *il faut avouer* that they may manage these matters differently in France. We should more freely indulge in double-shotted epithets of vituperation, had we not surmises of their liability to act inversely, like prosecutions for witchcraft of yore, which but tended to add to the number of *soi-disant* witches. We doubt the frequency of extreme cases of the vice in question. But there is no case in which severe psychological penalties are not incurred, those cases not excepted in which it is not a question of moral consideration at all : and there are such, as we shall show. "Children of two or three years of age," says Lallemand, "have been affected with priapism," (in their cases, of course, most inculpably) "owing to the irritation produced by ascarides." "Such children must consequently possess a like irresistible tendency to relieve this irritation as they have under the same complaint to scratch and rub the nose ; the sensations thus produced being calculated to lead to the formation of mischievous habits." He authenticates an account of a nurse who regularly employed certain means, to cause an infant to go to sleep, and who made no secret of those means and thought no harm of them. It follows that many in after life have bitterly reproached themselves for this malpractice as having thus furnished an almost supernatural demonstration of early and gratuitous depravity, and have attributed all their sufferings and misfortunes to this fatal habit. Self-abuse is in such cases only an intermediate link in the chain of causes and effects. But whatever the innocence or culpability of the party, there is no mistake with regard to the amount of mental suffering endured. The earlier the age at which this malpractice is ascertained to have commenced, the more probable will it be that ascarides, or stone, or other source of mechanical pressure, or of local irritation has originated it. We have the record of many cases by M. Lallemand, and of one by Mr. McDougall, in which the expulsion of ascarides was followed by rapid and complete recovery. It is met with as a sequel to blenorrhœa. There are other causes of this species of malpractice which, at least as causes, are out of the patient's control: congenital predisposition from redundancy and tenseness of the prepuce and other causes; congenital malconformation; cerebro-spinal disorder and disease; the action of certain medicines; spermatorrhœa may originate in these, may also be produced by intemperance, and this irrespectively of genital malpractices. It will have been seen that persons have embittered their lives by self-reproaches, whose self-reproaches have not been at all, or at least not darkly, deserved. Self-reproach, indeed, seems to be the natural retribution of self-abuse; whether merited or not, to be an ever attendant symptom of it. We have in Rousseau an example of genius blazing away as in a light-house to warn us away from the rocks and quicksands on which so many have perished. Lallemand is, after all, his only true commentator.

We have thus far spoken, for the most part, of cases exempt, wholly or in great part, from moral censure; but no ingenuity in seeking allowances will enable us to speak of all cases as such; we cannot speak of the retribution with which they meet as otherwise than deserved. There is self-contempt for pursuing an irrational course of misconduct —a reproving conscience for having done what is known to be wrong. Nothing that we could say would equal the self-taught eloquence of outraged nature. We might take hundreds of passages from Rousseau; but we abstain from this to quote rather the *ipsissima verba* of a sufferer of a later date. We quote it from case 32, p. 160, of Mr. McDougall's translation of Lallemand:

"At first," the patient says, "I felt a gradually increasing disgust of everything, and a constant sense of *ennui*. From that period I saw only the dark side of life. Thoughts of suicide soon afterwards occurred to me, and this state of mind continued for twelve months; after which, other ideas took the place of those respecting suicide. I considered myself the subject of ridicule, and fancied that the expression of my countenance, or my manner, excited an insulting gaiety in the persons I met. This notion each day acquired new strength; and often when in the street, or even when in my own house, or in a room surrounded by my relations and friends, I fancied I heard insults which were aimed at me,—*I think so still.* At length, as my state became worse, I thought every one insulted me, and *I still think so.* * * Wrapped up in my thoughts, I am indifferent to all external impressions. These signs are evidently those of imbecility. I admit that I might have had—that I may even now have *hallucinations,* but I am fully persuaded that these ideas are not without foundation; I am convinced that the expression of my countenance has something strange in it,—that people read in my looks the fears which agitate, and the thoughts which torment me,—and that they laugh at this unhappy weakness of intellect, which they ought rather to pity."

This is by no means a rare specimen; it is the language of the disease. There are the moral and mental pangs of hypochondriasis; there are propensities to suicide neutralized by a cowardice which renders the crime of *felo de se,* as it were, an incompleted tissue of perpetual guilt; there is a fearful looking forward to the future, both here and hereafter. What can be more lamentable? Cauterization in curing spermatorrhœa banishes all these sensations. There is a derangement of one of the wheels of the machinery, which being set right all goes on again correctly. Its symptoms amount to insanity, but the seat of the insanity is not the brain, but the prostate gland and orifices of the ductus seminales.

From abuse prior to puberty, after puberty—abuse succeeded by, abuse not succeeded by, spermatorrhœa, from spermatorrhœa however caused—result dejection of spirits, deterioration of the faculties of the mind. All these causes leave like mental results; the same in kind, differing only in degree. All produce greater exhaustion of the *vis vitæ* than natural excesses. When children naturally quick, become dull, the cause should be sought: the same with regard to students who have arrived at the age of puberty, and who, having been rapidly proficient, manifest a debility of the mental powers, and loiter on the road to academical distinction. When we miss the clearness of the decantered wine of youth, we must investigate the quality of the dregs, and seek the means of re-precipitating them. Where there are not great abilities, there are, in childhood and youth, at least physical energies. There is always something suspicious about old heads placed on young shoulders. Is unusual sedateness a sign of premature development of mind or of premature decrepitude? It may be either. We have sometimes observed a precocity that has been short-lived, succeeded by a state of mental imbecility that has been abiding. A fondness for solitude and exhibition of timidity in boys who have been bold and rackety, demands a parent's or preceptor's scrutiny.

The results of abuse, however intermediately physical, are eventually and mainly psychological. It is through the dark passes of self-abuse that many arrive at the bourne of confirmed insanity, whence, alas! the travellers that return, though more numerous than once, owing to better treatment, are not many.

Caustic will cure spermatorrhœa: to cure this is to cure a kind of insanity. But this being conceded, we still have a moral and spiritual, as well as physical being; the former of which apprises us feelingly of its existence when we violate the laws which should regulate it. The cases in which, whether as being persisted in or relinquished, this malpractice shows itself to be a matter of volition, are numerous. Let us then observe what it is and is not, theologically. No express command is violated, as in infractions of the seventh commandment; nor, as in certain other trespasses of a normal kind, physically considered, any direct precept. The crime of a certain offender mentioned in the Pentateuch, was not abuse as abuse: the gist of his offence consisted in his evasion of the prescribed obligation of raising offspring to perpetuate the memory and succeed to the property of a deceased relative, as was the custom of the times in which he lived. It comes, however, under the category of effeminacy, as condemned in the New Testament. Besides this, both nature and conscience tell the perpetrator that he is doing wrong; the sense of wrong doing is not slight, it is grievous and painful,—shame attends it. The psychological retribution is also severe; indeed, equally so with the moral. A species of isolation of heart and intellect ensues. In so far as pleasures are unshared, they are selfish. The whole spirit and genius of Christianity condemns selfishness. The mind, if not body, becomes emasculated. There is either existing, or apprehended, genital incapacity. A sense of segregation pervades his mind, affects his prospects in life; for "those who would have friends must show themselves friendly;" he, wrapped up in himself, feels and shows no such amities as all should cherish and all value. The ties seem severed which unite him to his species; he proceeds through life in a wild, dark path of his own choosing, beset with spectres and shadows and unfriendly faces, knowing no real comradeship except the evil and gloomy company of his own thoughts. It does not commonly —not frequently, induce spermatorrhea. The physical ill consequences of this malpractice are, in point of fact, nine times out of ten, next to none; but in all cases its moral and psychological consequences are marked and manifold; and woe to the one among the ten upon whom, physically speaking, falls the lot of decimation. There is nothing really contradictory in what we have advanced: passages which may seem so, admit of being honestly reconciled. It is always a monomania. Is it in any one given case culpably such or inculpably? The decision is often difficult. Science may sometimes pronounce the true verdict: in a majority of instances it lies out of its power and province to anticipate the decisions of a higher tribunal.

To recapitulate:—the malpractice alluded to is an offence against nature; it is, if not directly hostile to any command or precept of religion, an offence against the spirit of Christianity, were it only inasmuch as it is a singularly selfish vice; it is not visited, except in unusual or extreme cases, with physical retribution; its moral and psychological retribution is invariably severe; it effeminates and throws open the mind to the aggressions of empiricism; exposes it to the delusions of Superstition; it isolates the senses, affections, and ideas: thus isolating them, it alienates the perpetrator from society, rendering at once precarious and disadvantageous his position in the social scale. Its usual cure is the full and active employment of the physical and mental faculties: a growing enlightenment of mind which renders visible its evil tendencies; the formation and auspicious progress and favourable termination of some virtuous attachment. Speaking of its usual course and cure, more than this need not be, in fact, cannot be said of it truly,—and, alas! that we must add, the possession and *prestige* of genius supply neither charm nor periapt against it. On the contrary, its temptations peculiarly beset the studious, the recluse, and the imaginative.

We cannot condense into one hour's reading a sketch of a work which it would require a twelvemonth thoroughly to study, and which is an epitome of fourteen years' observation and experience. We may, on some future occasion, when more minutely tracking some of the highways and byways of the new territories to which M. Lallemand has pioneered the way, quote from his work more largely. It will be found a work of much interest to the consulting surgeon. It did not come within our province in this paper to treat it surgically. It nevertheless is certain that there are cases in which we may confidently have recourse to Surgery to cure Insanity. In all doubtful cases it should be ascertained whether spermatorrhœa exists.

There is a question put by Audrey in "As you like it," which often occurs to us as both pertinent and amusing: she asks, "Is it a true thing?" Well, supposing the reader to put the same question, we answer, we believe all we have said to be *pro tanto* true. We believe spermatorrhœa to be "a true thing." We, must, nevertheless, avoid being misled by a fourteen years' accumulation of cases into one volume by a surgeon who has acquired the position of a medical referee in such cases, into believing them more frequent than they really are. It is said of Sale, the translator of the Koran, that he became, through his absorption in his task, more than half a Mahometan. We knew, and have dined with Taylor, the translator of Plotinus, who was said to have erected in a room in his house (but we do not believe it) an altar to Jupiter. What is true is that the ethics of the works he translated effected a metastasis into his own mind, rendering him the most remarkable modern antique of his age. We must avoid the errors of both the Greek scholar and the Orientalist. We must not so enter into even a scientific pursuit as to suffer it to impose on us.

